# Graft rejection episodes after keratoplasty in Japanese eyes

**DOI:** 10.1038/s41598-023-29659-w

**Published:** 2023-02-14

**Authors:** Haguku Wajima, Takahiko Hayashi, Akira Kobayashi, Tsubasa Nishino, Natsuko Mori, Hideaki Yokogawa, Satoru Yamagami, Kazuhisa Sugiyama

**Affiliations:** 1 Department of Ophthalmology, Kanazawa University Hospital, Kanazawa University Graduate School of Medical Science, Takara-machi13-1, Kanazawa, Ishikawa 920-8641 Japan; 2 Department of Ophthalmology, Nihon University Itabashi Hospital, Tokyo, Japan; 3 Department of Ophthalmology, Yokohama Minami Kyousai Hospital, Yokohama, Japan

**Keywords:** Medical research, Transplantation

## Abstract

We aimed to investigate the clinical characteristics and risk factors for graft rejection after keratoplasty in Japanese patients. We enrolled 730 cases (566 patients) of penetrating keratoplasty (PK, N = 198), Descemet’s stripping automated endothelial keratoplasty (DSAEK, N = 277), non-Descemet’s stripping automated endothelial keratoplasty (nDSAEK, N = 138), and Descemet membrane endothelial keratoplasty (DMEK, N = 117). The incidence, clinical characteristics, and possible risk factors for graft rejection were analyzed. Graft rejection occurred in 65 cases (56 patients, 8.9%). The incidence rate of rejection was highest with PK (3.45/100 person-years), followed by DSAEK (2.34), nDSAEK (1.55), and DMEK (0.24). Cox regression analysis revealed keratoplasty type, younger age, indications (such as failed keratoplasty and infection), and steroid eyedrop use as possible risk factors. In the multivariate model adjusting baseline characteristics, PK and DSAEK had significantly higher hazard ratios (HRs) than DMEK (HR = 13.6, 95% confidence interval [CI] [1.83, 101] for PK, 7.77 [1.03, 58.6] for DSAEK). Although not statistically significant, the HR estimate of nDSAEK to DMEK (HR = 7.64, 95% CI [0.98, 59.6]) indicated higher HR in nDSAEK than in DMEK. DMEK is the favorable option among the four surgical procedures to avoid graft rejection after keratoplasty.

## Introduction

Over the past two decades, new lamellar keratoplasties, including Descemet stripping automated endothelial keratoplasty (DSAEK) and Descemet membrane endothelial keratoplasty (DMEK), have been developed to avoid complications caused by conventional penetrating keratoplasty (PK)^1–8^. Additionally, we^9^ and others^10,11^ eliminated Descemet stripping for bullous keratopathies in non-Fuchs type corneal endothelial dystrophy and referred to the modified procedure as non-Descemet Stripping Automated Endothelial Keratoplasty (nDSAEK)^9^. Moreover, both DSAEK and nDSAEK have proven quite effective for endothelial dysfunction, with rapid visual recovery and minimal induced astigmatism^9,12,13^.

Although PK remains a viable option^14^, the number of DSAEK and DMEK procedures has dramatically increased in the past two decades, and the number of DSAEK and DMEK procedures was almost comparable by the end of 2021 in the U.S.^15^. Recently, we reported a significant reduction in PK procedures while DSAEK/nDSAEK and DMEK procedures have significantly increased in our hospital^16,17^. Although these modern lamellar keratoplasties may reduce the incidence of immunological corneal graft rejection episodes, graft rejection and subsequent graft failure still occur, even after lamellar surgeries^18^. However, the incidence and characteristics of graft rejection after nDSAEK remain unclear.

This study aimed to investigate the clinical characteristics, risk factors, and probability of immunological graft rejection after keratoplasties, including PK and other novel lamellar keratoplasties (DSAEK/nDSAEK and DMEK), in Japanese patients.

## Results

Table 1 shows the demographics and comorbidities for the total and each surgical procedure of keratoplasty (N = 730). This study included data of 730 cases (359 males, 371 females; mean age ± standard deviation [SD], 71.2 ± 10.6 years) in 566 patients that underwent PK (N = 198), DSAEK (N = 277), nDSAEK (N = 138), and DMEK (N = 117). The mean and median postoperative duration were 1559 and 1190 days, respectively (90–5758 days). Background comorbidities of all keratoplasty cases included hypertension (N = 149, 20.4%), diabetes mellitus (N = 99, 13.6%), atopic dermatitis (N = 11, 1.5%), previous herpetic keratitis (N = 42, 5.8%), pre-existing glaucoma (N = 216, 29.6%), and prior keratoplasty in the opposite eye (N = 157, 21.5%). Significant heterogeneity among surgical procedures was observed for age, sex, hypertension, diabetes mellitus, herpetic keratitis, glaucoma, filtering bleb, and follow-up period up to the last visit.

**Table 1:** Demographics and comorbidities by surgical procedure of keratoplasty.

Characteristic	Total N = 730	Surgical procedure of keratoplasty				p-value^1^
		[A] PK N = 198	[B] DSAEK N = 277	[C] nDSAEK N = 138	[D] DMEK N = 117	
Number of patients, n	567	165	228	123	108	
Age [years]						
Mean (SD)	71.2 (10.6)	67.2 (12.3)	72.5 (9.8)	73.1 (10.2)	72.6 (8.1)	** < 0.001**
Median [range]	73.0 [41.0, 97.0]	70.0 [41.0, 90.0]	74.0 [41.0, 97.0]	74.0 [45.0, 90.0]	74.0 [48.0, 89.0]	
Sex, n (%)						
[1] Male	359 (49.2%)	119 (60.1%)	145 (52.3%)	56 (40.6%)	39 (33.3%)	** < 0.001**
[2] Female	371 (50.8%)	79 (39.9%)	132 (47.7%)	82 (59.4%)	78 (66.7%)	
Hypertension, n (%)	149 (20.4%)	32 (16.2%)	69 (24.9%)	36 (26.1%)	12 (10.3%)	**0.003**
Diabetes mellitus, n (%)	99 (13.6%)	21 (10.6%)	52 (18.8%)	18 (13.0%)	8 (6.8%)	**0.014**
Atopic dermatitis, n (%)	11 (1.5%)	5 (2.5%)	6 (2.2%)	0 (0.0%)	0 (0.0%)	0.192
Herpetic keratitis, n (%)	42 (5.8%)	27 (13.6%)	9 (3.2%)	5 (3.6%)	1 (0.9%)	** < 0.001**
Glaucoma and surgery, n (%)						
[1] No glaucoma	514 (70.4%)	150 (75.8%)	158 (57.0%)	98 (71.0%)	108 (92.3%)	** < 0.001**
[2] Glaucoma without surgery	64 (8.8%)	14 (7.1%)	27 (9.7%)	15 (10.9%)	8 (6.8%)	
[3] Glaucoma with surgery	152 (20.8%)	34 (17.2%)	92 (33.2%)	25 (18.1%)	1 (0.9%)	
Prior KP in opposite eye, n (%)						
[1] No	572 (78.4%)	158 (79.8%)	222 (80.1%)	113 (81.9%)	79 (67.5%)	0.120
[2] Yes	157 (21.5%)	39 (19.7%)	55 (19.9%)	25 (18.1%)	38 (32.5%)	
[3] Unknown	1 (0.1%)	1 (0.5%)	0 (0.0%)	0 (0.0%)	0 (0.0%)	
Filtering bleb, n (%)	127 (17.4%)	25 (12.6%)	80 (28.9%)	22 (15.9%)	0 (0.0%)	** < 0.001**
Follow-up period up to last visit [days]						
Mean (SD)	1,559 (1,197)	1,949 (1,446)	1,173 (852)	1,992 (1,399)	1,307 (714)	** < 0.001**
Median [range]	1,190 [90, 5,758]	1,490 [97, 5,758]	948 [90, 5,632]	1,854 [95, 5,198]	1,123 [165, 3,856]	
Follow-up period up to last visit [years]						
Mean (SD)	4.3 (3.3)	5.3 (4.0)	3.2 (2.3)	5.5 (3.8)	3.6 (2.0)	
Median [range]	3.3 [0.2, 15.8]	4.1 [0.3, 15.8]	2.6 [0.2, 15.4]	5.1 [0.3, 14.2]	3.1 [0.5, 10.6]	

*DMEK* descemet membrane endothelial keratoplasty, *(n)DSAEK* (non-)descemet’s stripping automated endothelial keratoplasty, *PK* penetrating keratoplasty, *SD* standard deviation.

Significant values are given in bold.

^1^Kruskal–Wallis test for continuous variables; Chi-square test for categorical variables.

Table 2 summarizes the indications for the total and each surgical procedure of keratoplasty (N = 730). Failed keratoplasty was the most common indication for keratoplasty throughout the 15-year period (N = 172, 23.6%), followed by argon laser iridotomy (N = 125, 17.1%), pseudophakic bullous keratopathy (N = 82, 11.2%), glaucoma surgery (N = 78, 10.7%), corneal opacity (N = 48, 6.6%), Fuchs corneal endothelial dystrophy (N = 48, 6.6%), exfoliation syndrome (N = 27, 3.7%), keratoconus (N = 21, 2.9%), perforation (N = 17, 2.3%), infection (N = 13, 1.8%), cytomegalovirus corneal endotheliitis (N = 12, 1.6%), corneal dystrophy/degeneration (N = 11, 1.5%), iridocorneal endothelial syndrome (N = 3, 0.4%), and other causes (N = 73, 10.0%). Failed PK was the most common cause of failed keratoplasty (N = 67, 39.0%), followed by failed DSAEK (N = 67, 39.0%), failed nDSAEK (N = 17, 9.9%), failed DMEK (N = 15, 8.7%), and failed deep anterior lamellar keratoplasty (DALK) (N = 5, 2.9%). The distribution of indications was significantly heterogeneous among surgical procedures.

**Table 2:** Indication of keratoplasty.

Characteristic	Total N = 730	Surgical procedure of keratoplasty				p-value^1^
		[A] PK N = 198	[B] DSAEK N = 277	[C] nDSAEK N = 138	[D] DMEK N = 117	
Indication, n (%)						
[01] Failed KP	172 (23.6%)	44 (22.2%)	95 (34.3%)	26 (18.8%)	7 (6.0%)	** < 0.001**
[02] ALI	125 (17.1%)	4 (2.0%)	33 (11.9%)	47 (34.1%)	41 (35.0%)	
[03] PBK	82 (11.2%)	6 (3.0%)	34 (12.3%)	26 (18.8%)	16 (13.7%)	
[04] Glaucoma surgery	78 (10.7%)	14 (7.1%)	48 (17.3%)	16 (11.6%)	0 (0.0%)	
[05] Corneal opacity	48 (6.6%)	40 (20.2%)	7 (2.5%)	0 (0.0%)	1 (0.9%)	
[06] FED	48 (6.6%)	0 (0.0%)	17 (6.1%)	3 (2.2%)	28 (23.9%)	
[07] XFS	27 (3.7%)	1 (0.5%)	11 (4.0%)	6 (4.3%)	9 (7.7%)	
[08] Keratoconus	21 (2.9%)	21 (10.6%)	0 (0.0%)	0 (0.0%)	0 (0.0%)	
[09] Perforation	17 (2.3%)	17 (8.6%)	0 (0.0%)	0 (0.0%)	0 (0.0%)	
[10] Infection	13 (1.8%)	13 (6.6%)	0 (0.0%)	0 (0.0%)	0 (0.0%)	
[11] CMV	12 (1.6%)	0 (0.0%)	7 (2.5%)	1 (0.7%)	4 (3.4%)	
[12] Corneal dystrophy/degeneration	11 (1.5%)	7 (3.5%)	1 (0.4%)	0 (0.0%)	3 (2.6%)	
[13] ICE	3 (0.4%)	1 (0.5%)	2 (0.7%)	0 (0.0%)	0 (0.0%)	
[14] Others	73 (10.0%)	30 (15.2%)	22 (7.9%)	13 (9.4%)	8 (6.8%)	
Failed KP breakdown, n						
[1] Failed PK	67	36	14	15	2	
[2] Failed DSAEK	67	5	58	4	0	
[3] Failed nDSAEK	17	0	9	6	2	
[4] Failed DMEK	15	0	12	0	3	
[5] Failed DALK	5	3	2	0	0	
[6] Unknown	1	0	0	1	0	

*ALI* argon laser iridotomy, *CMV* cytomegalovirus, *DALK* Deep anterior lamellar keratoplasty, *DMEK* Descemet membrane endothelial keratoplasty, *(n)DSAEK* (non-)Descemet’s stripping automated endothelial keratoplasty, *FED* Fuchs’ endothelial dystrophy, *ICE* iridocorneal endothelial syndrome, *PBK* pseudophakic bullous keratopathy, *PK* penetrating keratoplasty, *XFS* exfoliation syndrome.

Significant values are given in bold.

^1^Chi-square test.

Table 3 summarizes the combined surgery and graft size for the total and each keratoplasty procedure (N = 730). Among all keratoplasty cases enrolled in this study, 603 cases (82.6%) were simple keratoplasty procedures, and the remaining (N = 127,17.4%) had simultaneous surgeries at the time of keratoplasty, including phacoemulsification with intraocular lens implantation and other procedures. Median graft size for PK, DSAEK, nDASEK, and DMEK were 7.75 (range: 5.5–8.75) mm, 8.0 (range: 6.0–8.5) mm, 8.0 (range: 6.0–8.5) mm, and 8.0 (range: 7.25–8.5) mm, respectively. Graft size was significantly heterogenous among the surgical procedures.

**Table 3:** Combined surgery and graft size of keratoplasty.

Characteristic	Total N = 730	Surgical procedure of keratoplasty				p-value^1^
		[A] PK N = 198	[B] DSAEK N = 277	[C] nDSAEK N = 138	[D] DMEK N = 117	
Simple vs. Combined KP, n (%)						
[1] Simple keratoplasty	603 (82.6%)	160 (80.8%)	230 (83.0%)	113 (81.9%)	100 (85.5%)	0.878
[2] Combined keratoplasty	127 (17.4%)	38 (19.2%)	47 (17.0%)	25 (18.1%)	17 (14.5%)	
Combined surgery breakdown, n						
[1] Cataract surgery	102	27	35	23	17	
[2] IOL fixaction	20	8	10	2	0	
[3] Anterior vitrectomy	2	2	0	0	0	
[4] Pupiloplasty	2	0	2	0	0	
[5] KLAL	1	1	0	0	0	
Graft size [mm]						
Mean (SD)	7.77 (0.44)	7.63 (0.42)	7.79 (0.50)	7.80 (0.43)	7.95 (0.25)	** < 0.001**
Median [Range]	8.00 [5.50, 8.75]	7.75 [5.50, 8.75]	8.00 [6.00, 8.50]	8.00 [6.00, 8.50]	8.00 [7.25, 8.50]	
Graft size group, n (%)						
[1] 4.0–< 7.6 mm	195 (26.7%)	66 (33.3%)	79 (28.5%)	36 (26.1%)	14 (12.0%)	
[2] 7.6–< 7.8 mm	128 (17.5%)	105 (53.0%)	4 (1.4%)	5 (3.6%)	14 (12.0%)	
[3] 7.8–< 8.1 mm	316 (43.3%)	18 (9.1%)	155 (56.0%)	80 (58.0%)	63 (53.8%)	
[4] 8.1–9.0 mm	57 (7.8%)	5 (2.5%)	27 (9.7%)	6 (4.3%)	19 (16.2%)	
[5] Unknown	34 (4.7%)	4 (2.0%)	12 (4.3%)	11 (8.0%)	7 (6.0%)	

*DMEK* descemet membrane endothelial keratoplasty, *(n)DSAEK* (non-)descemet’s stripping automated endothelial keratoplasty, *KLAL* keratolimbal allograft, *PK* penetrating keratoplasty, *SD* standard deviation.

Significant values are given in bold.

^1^Chi-square test for ‘Simple vs. Combined KP’; Kruskal–Wallis test for ‘Graft size’.

*For cases with multiple concomitant techniques, the most representative technique was selected.

^♰^*ECCE* extracapsular cataract extraction, *ICCE* intracapsular cataract extraction, *IOL* intraocular lens.

Table 4 summarizes the graft rejection episodes, eye drops at rejection or last visit, and graft outcomes in all cases (N = 730). Overall, graft rejection episodes occurred in 65 cases in 56 patients (8.9%). PK showed the highest rejection rate (33/198, 16.7%), followed by nDSAEK (11/138, 8.0%), DSAEK (20/277, 7.2%), and DMEK (1/117, 0.9%). The mean follow-up period for all patients (N = 730) from the time of surgery to the time of rejection or last visit (for patients without rejection) was 1468 days. At the time of rejection or last visit, the following 627 cases (85.9%) used steroid eye drops: PK (N = 163, 82.3%), DSAEK (N = 246, 88.8%), nDSAEK (N = 103, 74.6%), and DMEK (N = 115, 98.3%). Among steroid eye drops administered, betamethasone was used in 164 cases (26.2%), and fluorometholone was used in the remaining 463 cases (73.8%). Regarding the graft outcomes in all cases (N = 730), 574 cases (78.6%) remained clear, while the remaining cases (N = 156, 21.4%) failed. Subgroup analysis of the surgical procedure of keratoplasty and clear graft without rejection were observed in PK (N = 111, 56.1%), DSAEK (N = 204, 73.6%), nDSAEK (N = 101, 73.2%), and DMEK (N = 108, 92.3%). Significant heterogeneity among surgical procedures was observed for rejection episode, follow-up period for rejection, eye drop usage, and graft outcome.

**Table 4:** Eye drop at rejection (or last visit) and graft outcome after keratoplasty in all cases (N = 730).

Characteristic	Total N = 730	Surgical procedure of keratoplasty				p-value^1^
		[A] PK N = 198	[B] DSAEK N = 277	[C] nDSAEK N = 138	[D] DMEK N = 117	
Rejection episode, n (%)	65 (8.9%)	33 (16.7%)	20 (7.2%)	11 (8.0%)	1 (0.9%)	** < 0.001**
Follow-up period for rejection [days]						
Mean (SD)	1,468 (1,184)	1,764 (1,460)	1,126 (860)	1,875 (1,386)	1,295 (714)	** < 0.001**
Median [range]	1,138 [5, 5,758]	1,301 [26, 5,758]	888 [5, 5,632]	1,728 [95, 5,198]	1,115 [165, 3,856]	
Follow-up period for rejection [years]						
Mean (SD)	4.0 (3.2)	4.8 (4.0)	3.1 (2.4)	5.1 (3.8)	3.5 (2.0)	
Median [range]	3.1 [0.0, 15.8]	3.6 [0.1, 15.8]	2.4 [0.0, 15.4]	4.7 [0.3, 14.2]	3.1 [0.5, 10.6]	
Eye drop usage at rejection (or last visit), n (%)						
[1] No	103 (14.1%)	35 (17.7%)	31 (11.2%)	35 (25.4%)	2 (1.7%)	** < 0.001**
[2] Yes	627 (85.9%)	163 (82.3%)	246 (88.8%)	103 (74.6%)	115 (98.3%)	
Eye drop breakdown, n						
[1] Fluorometholone	463	103	168	82	110	
[2] Betamethasone	164	60	78	21	5	
Graft outcome, n (%)						
[1] Clear	524 (71.8%)	111 (56.1%)	204 (73.6%)	101 (73.2%)	108 (92.3%)	** < 0.001**
[2] Recovered	50 (6.8%)	23 (11.6%)	17 (6.1%)	9 (6.5%)	1 (0.9%)	
[3] Failed	156 (21.4%)	64 (32.3%)	56 (20.2%)	28 (20.3%)	8 (6.8%)	

*DMEK* descemet membrane endothelial keratoplasty, *(n)DSAEK* (non-)descemet’s stripping automated endothelial keratoplasty, *PK* penetrating keratoplasty, *SD* standard deviation.

Significant values are given in bold.

^1^Chi-square test for categorical variables; Kruskal–Wallis test for ‘Follow-up period for rejection’.

**Figure 1:**
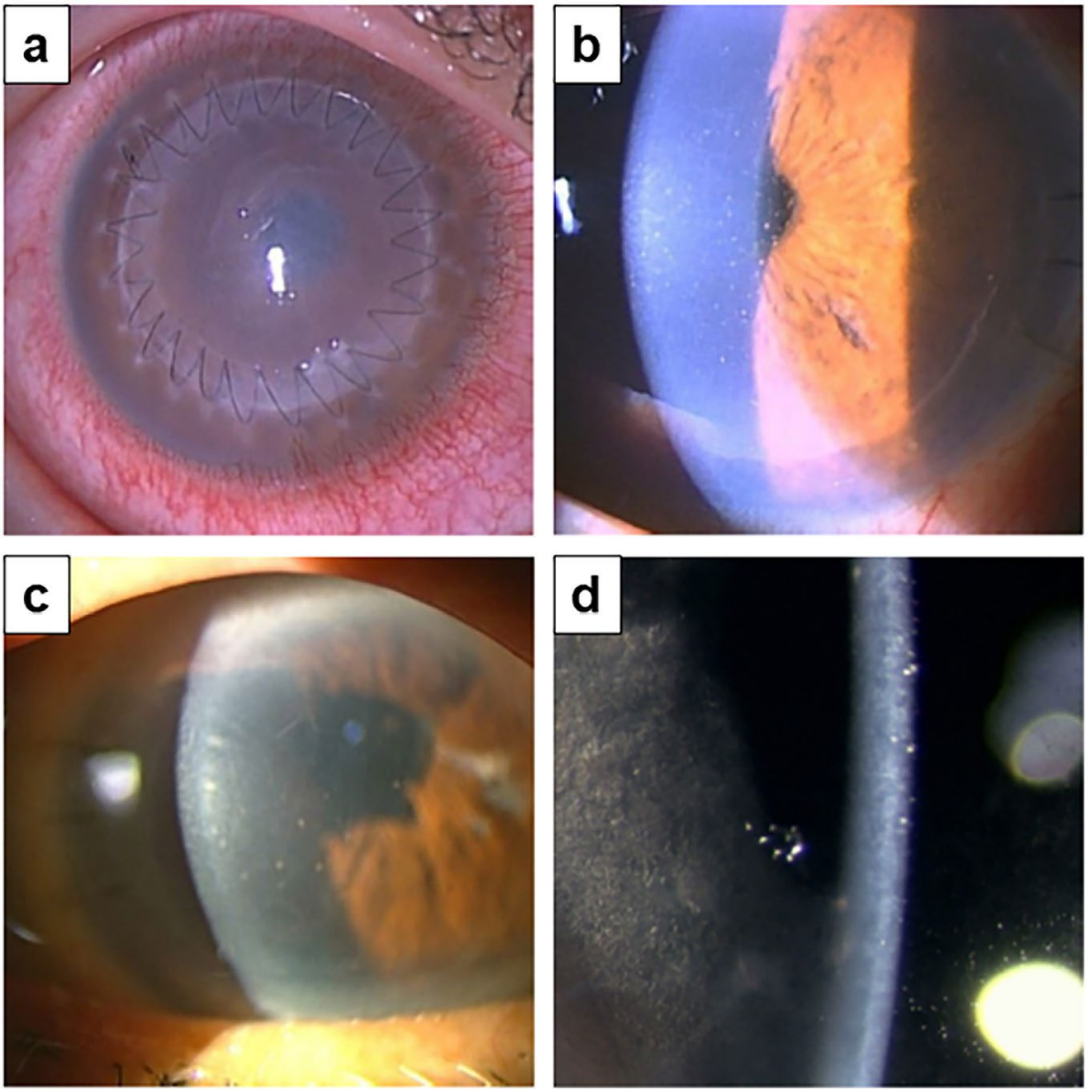
Representative slit-lamp photos of graft rejection after keratoplasty. (**a**) Graft rejection after PK for keratoconus in an 18-year-old male patient. The patient had been treated with 0.1% betamethasone eyedrop at the onset of rejection. Endothelial rejection with decreased visual acuity, conjunctival hyperemia, corneal edema, and keratic precipitates were observed 214 days postoperatively. Additionally, despite topical and oral steroid therapy, the graft failed. (**b**) Graft rejection after DSAEK for bullous keratopathy due to trabeclectomy in a 67-year-old male patient. Endothelial rejection with decreased visual acuity, conjunctival hyperemia, and keratic precipitates with pigments were observed after 164 days postoperatively. He discontinued 0.1% fluorometholone eye drop one month before. The rejection was cured with steroid eyedrop after one month. (**c**) Graft rejection after nDSAEK for PBK in a 75-year-old female patient. Despite betamethasone eye drops treatment after surgery, rejection occurred on day 138 postoperatively. The patient had decreased vision, corneal edema, and keratic precipitates, rather than conjunctival hyperemia. Topical and oral steroid treatment improved corneal clarity and rejection reaction after 3 weeks. (**d**) Graft rejection after DMEK for bullous keratopathy due to argon laser iridotomy in a 62-year-old female patient. Endothelial rejection with decreased visual acuity, conjunctival hyperemia, and keratic precipitates with pigments were observed 2031 days postoperatively. She used 0.1% fluorometholone eye drop at the time of rejection. The rejection was cured with a steroid eyedrop after one month. *DMEK* descemet membrane endothelial keratoplasty, *DSAEK* descemet’s stripping automated endothelial keratoplasty, *nDSAEK* non-Descemet’s stripping automated endothelial keratoplasty, *PK* penetrating keratoplasty, *PBK* pseudophakic bullous keratopathy.

Table 5 summarizes findings on rejection episodes and graft outcomes in limited cases with a rejection episode (N = 65). The mean onset of rejection after overall keratoplasty was 641 ± 670 (days ± SD): PK (717 ± 735 days), DSAEK (507 ± 460 days), nDSAEK (661 ± 829 days), and DMEK (629 days). Figure 1 shows representative slit-lamp photographs of the graft rejection for each keratoplasty procedure. As shown in Table 5, 17 of 33 PK rejection cases (51.5%), 8 of 20 DSAEK rejection cases (40.0%), 7 of 11 nDSAEK rejection cases (63.6%), and 1 of 1 DMEK rejection case (100%) were symptomatic. Signs of immunological rejection at the initial diagnosis in PK, DSAEK, nDSAEK, and DMEK included conjunctival hyperemia (27.3%, 30.0%, 18.2%, and 0%, respectively), diffuse corneal edema (45.5%, 45.0%, 45.5%, and 100%, respectively), and keratic precipitates (84.8%, 95.0%, 90.9%, and 100%, respectively). Although this study demonstrated rejection lines in four cases of PK, no endothelial rejection lines were observed in all types of endothelial keratoplasty. Most rejected grafts cleared after the episode (50, 76.9%); however, 10 eyes with rejection (30.3%) that underwent PK, 3 eyes with rejection (15.0%) that underwent DSAEK, and 2 eyes with rejection (18.2%) that underwent nDSAEK progressed to graft failure.

**Table 5:** Findings at rejection episode and graft outcome after the episode [Cases with rejection episode] (N = 65).

Characteristic	Cases with rejection N = 65	Surgical procedure of keratoplasty			
		[A] PK N = 33	[B] DSAEK N = 20	[C] nDSAEK N = 11	[D] DMEK N = 1
Time of onset from keratoplasty [days]					
Mean (SD)	641 (670)	717 (735)	507 (460)	661 (829)	629 (NA)
Median [range]	410 [5, 3149]	438 [26, 3149]	284 [5, 1408]	362 [132, 2994]	629 [629, 629]
Eye drop, n (%)					
[1] No	8 (12.3%)	1 (3.0%)	4 (20.0%)	3 (27.3%)	0 (0.0%)
[2] Fluorometholone	32 (49.2%)	14 (42.4%)	11 (55.0%)	6 (54.5%)	1 (100.0%)
[3] Betamethasone	25 (38.5%)	18 (54.5%)	5 (25.0%)	2 (18.2%)	0 (0.0%)
Suture related event, n (%)	12 (18.5%)	8 (24.2%)	3 (15.0%)	1 (9.1%)	0 (0.0%)
Symptomatic case, n (%)	33 (50.8%)	17 (51.5%)	8 (40.0%)	7 (63.6%)	1 (100.0%)
Conjunctival hyperemia, n (%)	17 (26.2%)	9 (27.3%)	6 (30.0%)	2 (18.2%)	0 (0.0%)
Corneal edema, n (%)	30 (46.2%)	15 (45.5%)	9 (45.0%)	5 (45.5%)	1 (100.0%)
Keratic precipitate, n (%)	58 (89.2%)	28 (84.8%)	19 (95.0%)	10 (90.9%)	1 (100.0%)
Vascularization of cornea, n (%)	13 (20.0%)	9 (27.3%)	3 (15.0%)	1 (9.1%)	0 (0.0%)
Peripheral anterior synechia, n (%)	9 (13.8%)	4 (12.1%)	4 (20.0%)	1 (9.1%)	0 (0.0%)
Rejection line, n (%)	4 (6.2%)	4 (12.1%)	0 (0.0%)	0 (0.0%)	0 (0.0%)
Graft outcome, n (%)					
[1] Recovered	50 (76.9%)	23 (69.7%)	17 (85.0%)	9 (81.8%)	1 (100.0%)
[2] Failed	15 (23.1%)	10 (30.3%)	3 (15.0%)	2 (18.2%)	0 (0.0%)

*DMEK* descemet membrane endothelial keratoplasty, *(n)DSAEK* (non-)descemet’s stripping automated endothelial keratoplasty, *PK* penetrating keratoplasty, *SD* standard deviation.

Table 6 summarizes the incidence rates of rejection episodes according to the time interval after each type of keratoplasty (N = 730). The overall incidence rates of rejection episodes [per 100 person-years] were the highest in PK (3.45), followed by DSAEK (2.34), nDSAEK (1.55), and DMEK (0.24) in descending order. In the DMEK group, only one rejection episode was observed, and the overall estimate of the rejection incidence rate was extremely low compared with other types of keratoplasty. In the other three groups, PK, DSAEK, and nDSAEK, the highest incidence rate was observed between 0 and 1 year after each keratoplasty.

**Table 6:** Incidence rate of rejection episode.

	Surgical procedure of keratoplasty			
	[A] PK	[B] DSAEK	[C] nDSAEK	[D] DMEK
Overall summary statistics				
Number of patients	198	277	138	117
Person-years [100 person-years]	9.57	8.54	7.09	4.15
Rejection episodes	33	20	11	1
Incidence rate [/100 person-years]	3.45	2.34	1.55	0.24
Incidence rate of rejection episodes by time period after keratoplasty (number of episodes/100 person-years)				
[1] 0–< 1 year after keratoplasty	7.55 (14/1.85)	4.22 (11/2.61)	4.56 (6/1.31)	0.00 (0/1.16)
[2] 1–< 2 years after keratoplasty	6.44 (10/1.55)	0.96 (2/2.09)	1.83 (2/1.09)	0.92 (1/1.08)
[3] 2–< 3 years after keratoplasty	0.78 (1/1.27)	2.83 (4/1.42)	2.21 (2/0.91)	0.00 (0/0.80)
[4] 3–< 4 years after keratoplasty	2.93 (3/1.03)	3.27 (3/0.92)	0.00 (0/0.77)	0.00 (0/0.46)
[5] 4–< 5 years after keratoplasty	1.26 (1/0.79)	0.00 (0/0.54)	0.00 (0/0.70)	0.00 (0/0.26)
[6] 5 + years after keratoplasty	1.30 (4/3.07)	0.00 (0/0.97)	0.44 (1/2.30)	0.00 (0/0.39)

*DMEK* descemet membrane endothelial keratoplasty, *(n)DSAEK* (Non-)Descemet’s stripping automated endothelial keratoplasty, *PK* penetrating keratoplasty.

**Figure 2:**
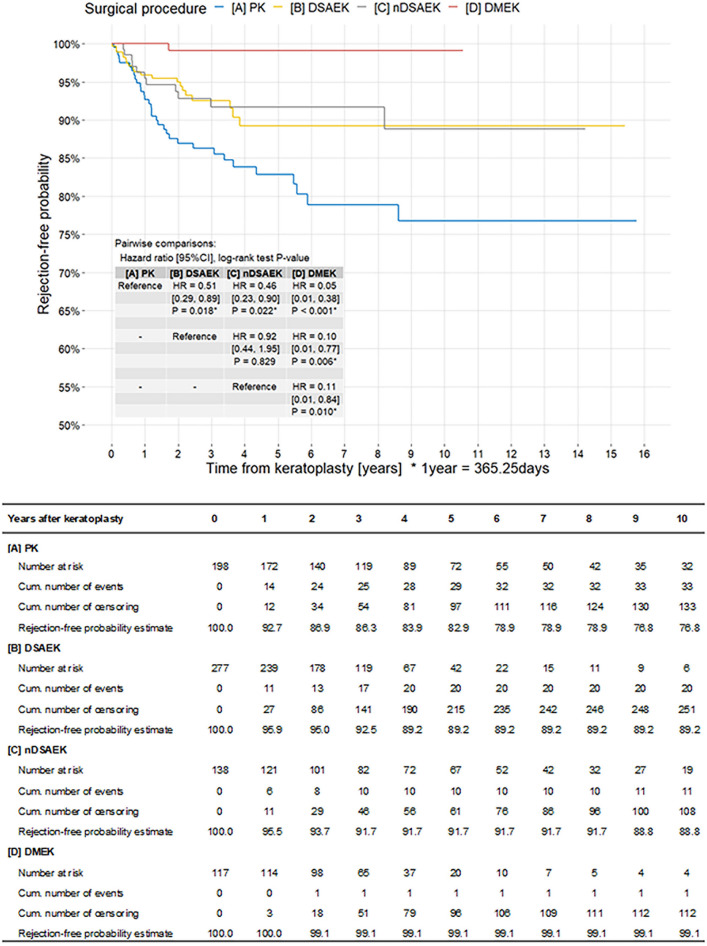
Kaplan–Meier survival curves of rejection episode by surgical procedure groups (N = 730). The PK group has the highest hazard estimate of rejection, with significant differences between this group and the other three groups (*P* = 0.018 for DSAEK, *P* = 0.022 for nDSAEK, and *P* < 0.001 for DMEK). No significant difference was observed between DSAEK and nDSAEK (HR = 0.92, 95% CI [0.44, 1.95], P = 0.829). The DMEK group has the lowest hazard estimate of rejection, with significant differences between this group and the other three groups (*P* < 0.001, PK; P = 0.006, DSAEK; and P = 0.010, nDSAEK). *DMEK* descemet membrane endothelial keratoplasty, *DSAEK* descemet’s stripping automated endothelial keratoplasty, *nDSAEK* non-descemet’s stripping automated endothelial keratoplasty, *PK* penetrating keratoplasty.

Figure 2 shows the Kaplan–Meier curve of rejection episodes by surgical procedure group (N = 730). The PK group had the highest hazard estimate of rejection, with significant differences between this group and the other three groups (*P* = 0.018, DSAEK; *P* = 0.022, nDSAEK; and *P* < 0.001, DMEK). Additionally, no significant difference was observed between DSAEK and nDSAEK (hazard ratio [HR] = 0.92, 95% confidence interval [CI] [0.44, 1.95], *P* = 0.829). The DMEK group had the lowest hazard estimate of rejection, and significant differences were observed between this group and the other three groups (*P* < 0.001, PK; *P* = 0.006, DSAEK; and *P* = 0.010, nDSAEK).

Table 7 shows the results of the univariate and multivariate Cox regression analyses of rejection episodes (N = 730). In the univariate model, surgical procedure, age, indications (failed keratoplasty and infection), and eye drop at rejection had significant effects on rejection episodes (*P* < 0.001, 0.005, 0.014, 0.021, and < 0.001, respectively). In the multivariate model with baseline characteristics, the surgical procedure, age, and indications (failed keratoplasty and infection) remained after backward variable selection. For surgical procedure, PK and DSAEK had significantly higher HR than DMEK (HR = 13.6, 95%CI [1.83, 101] for PK, 7.77 [1.03, 58.6] for DSAEK), and the HR estimate of nDSAEK to DMEK (HR = 7.64, 95%CI [0.98, 59.6]) indicated higher HR in nDSAEK than DMEK, although it was not statistically significant. Moreover, younger age, indication for failed keratoplasty, and infection were identified as risk factors at baseline (*P* = 0.023, 0.019, and 0.043, respectively). In the multivariate model with eye drops, significant effects of eye drops at rejection or last visit (*P* = 0.019) were observed after adjustment for baseline risk factors. Compared to eye drop non-users at rejection or last visit, a higher risk of rejection was observed in eye drop users (fluorometholone HR:1.37, 95% CI [0.63, 3.00] and betamethasone HR:2.68, 95% CI [1.19, 6.04] ).

**Table 7:** Univariate and multivariate Cox regression on rejection episode.

Characteristic	Univariate model			Multivariate model without eye drop			Multivariate model with eye drop		
	HR^1^	95% CI^1^	p-value	HR^1^	95% CI^1^	p-value	HR^1^	95% CI^1^	p-value
Surgical procedure									
[A] PK	19.0	2.60, 139	** < 0.001**	13.6	1.83, 101	**0.001**	12.3	1.64, 92.1	**0.003**
[B] DSAEK	9.66	1.30, 72.0		7.77	1.03, 58.6		6.82	0.90, 51.8	
[C] nDSAEK	8.71	1.12, 67.6		7.64	0.98, 59.6		7.55	0.96, 59.3	
[D] DMEK	1.00	–		1.00	–		1.00	–	
Age	0.72	0.58, 0.90	**0.005**	0.77	0.62, 0.96	**0.023**	0.78	0.62, 0.97	**0.032**
Sex									
[1] Male	1.00	–	0.235						
[2] Female	0.74	0.46, 1.21							
Hypertension	0.92	0.48, 1.76	0.805						
Diabetes mellitus	1.12	0.55, 2.26	0.756						
Herpetic keratitis	1.84	0.79, 4.27	0.189						
Glaucoma and surgery									
[1] No glaucoma	1.00	–	0.203						
[2] Glaucoma without surgery	0.55	0.17, 1.77							
[3] Glaucoma with surgery	1.44	0.82, 2.51							
Prior keratoplasty in opposite eye									
[1] No/unknown	1.00	–	0.147						
[2] Yes	1.50	0.88, 2.57							
Indication—[01] Failed keratoplasty	1.95	1.17, 3.24	**0.014**	1.92	1.13, 3.25	**0.019**	1.73	1.01, 2.96	0.052
Indication—[02] ALI	0.61	0.29, 1.29	0.171						
Indication—[03] PBK	0.39	0.12, 1.26	0.068						
Indication—[04] Glaucoma surgery	0.89	0.38, 2.06	0.782						
Indication—[05] Corneal opacity	1.76	0.84, 3.71	0.163						
Indication—[06] FED	0.39	0.10, 1.60	0.127						
Indication—[07] XFS	0.49	0.07, 3.57	0.432						
Indication—[08] Keratoconus	1.93	0.70, 5.31	0.248						
Indication—[09] Perforation	0.85	0.12, 6.11	0.866						
Indication—[10] Infection	4.24	1.54, 11.7	**0.021**	3.60	1.23, 10.5	**0.043**	2.86	0.96, 8.48	0.092
Indication—[11] CMV corneal endotheliitis	0.00	Not estimable	0.131						
Indication—[12] Corneal dystrophy/degeneration	0.94	0.13, 6.81	0.954						
Indication—[13] ICE	0.00	Not estimable	0.372						
Indication—[14] Others	0.61	0.22, 1.68	0.306						
Simple vs. combined surgery									
[1] Simple keratoplasty	1.00	–	0.433						
[2] Combined keratoplasty	0.76	0.38, 1.54							
Graft size									
[1] 4.0–< 7.8 mm	1.00	–	0.277						
[2] 7.6– < 8.1 mm	0.58	0.34, 0.99							
[3] 8.1–< 9.0 mm	0.77	0.29, 2.02							
[4] Unknown	0.68	0.21, 2.28							
Eye drop at rejection (or last visit)									
[1] No	1.00	–	** < 0.001**				1.00	–	**0.019**
[2] Fluorometholone	1.00	0.46, 2.17					1.37	0.63, 3.00	
[3] Betamethasone	2.99	1.34, 6.66					2.68	1.19, 6.04	

^1^*HR* hazard ratio, *CI* confidence interval.

*ALI* argon laser iridotomy, *CMV* cytomegalovirus, *DALK* deep anterior lamellar keratoplasty, *DMEK* descemet membrane endothelial keratoplasty, *(n)DSAEK* (non-)descemet’s stripping automated endothelial keratoplasty, *FED* Fuchs’ endothelial dystrophy, *ICE* iridocorneal endothelial syndrome, *PBK* pseudophakic bullous keratopathy, *PK* penetrating keratoplasty, *XFS* exfoliation syndrome.

Significant values are given in bold.

The results of subgroup analyses on rejection episodes by demographics, indication for keratoplasty, and simple/combined keratoplasty and graft size that correspond to Tables 1, 2, and 3 are summarized in Supplementary Tables S1–S3.

## Discussion

This study investigated the clinical characteristics, risk factors, and probability of immunological graft rejection after keratoplasty, including PK, DSAEK, nDSAEK, and DMEK in Asian patients. We showed that graft rejection episodes occurred in 65 of the 56 patients (65/730, 8.9%) who underwent keratoplasty. In the assessment of each keratoplasty technique, PK had the highest rejection rate (33/198, 16.7%) than other types of surgeries, followed by nDSAEK (11/138, 8.0%), DSAEK (20/277, 7.2%), and DMEK (1/117, 0.9%) (Table 4). For improved accuracy in statistical analysis, the incidence rates of rejection episodes were investigated using the person-years method. Consequently, similar outcomes were obtained; PK was the highest (3.45/100 person-years), followed by DSAEK (2.34), nDSAEK (1.55), and DMEK (0.24) in descending order (Table 6). Furthermore, Cox regression analysis of the HR between surgical groups showed that the PK group had the highest hazard estimate of rejection, and significant differences were observed between this group and the other three groups (*P* = 0.018, DSAEK; *P* = 0.022, nDSAEK; and *P* < 0.001, DMEK). Most notably, no significant difference was observed between DSAEK and nDSAEK (HR = 0.92, 95% CI = [0.44, 1.95], *P* = 0.829). The DMEK group had the lowest hazard estimate of rejection, and significant differences were observed between this group and the other three groups (*P* < 0.001, PK; *P* = 0.006, DSAEK; and *P* = 0.010, nDSAEK). These results are consistent with those of previous studies^19–25^, thus confirming the excellent advantages of a lower rejection rate of endothelial keratoplasties compared with conventional PK^25^; DMEK, in particular, showed the lowest incidence rate of graft rejection, clearly indicating the superiority of this procedure^19^. To the best of our knowledge, this study is the largest case series of recent keratoplasties in Japan.

The reasons for lower graft rejection after endothelial keratoplasty are as follows^26^: (a) The transplanted tissue is inserted into the anterior chamber and has no exposure to the surface, where antigen-presenting cells and antibodies are present. Hence, anterior chamber-associated immune deviation may be effective. (b) A significant reduction in the number of sutures connecting the host and donor tissues may lead to fewer suture-related rejection episodes. (c) The absence of direct contact between the host stromal vessels and transplanted tissue disrupts the immune effector and effector arcs. (d) Reduced immunogenicity of the donated tissue due to the absence of donor epithelium and the majority of the stroma.

Clinical outcomes of nDSAEK have been reported to be similar to those of DSAEK^9,12,13,27–33^. Several reports support the advantages of nDSAEK, particularly for failed PK, as stripping Descemet’s membrane of failed PK may cause PK wound separation or weakness^27–29^. The histology and in vivo observation of the residual host endothelial cells after nDSAEK have already been thoroughly studied, including decreased density, loss of pump function, apoptosis, and altered morphology in a rabbit model by in vivo confocal microscopy^34^. Additionally, in vivo laser confocal microscopy in human studies after nDSAEK can detect subclinical corneal abnormalities, including subepithelial haze, host-recipient interface haze, host stromal needle-shaped materials, and host-recipient interface particles with characteristic giant particles^35^. Using immunohistochemistry and transmission electron microscopy, 2 weeks after nDSAEK in a rabbit model, host endothelial cells appeared to be morphologically altered, occasionally detached from the adjacent Descemet membrane, extending into the graft stroma or engulfing strands of the grafted stroma at the interface^36^. However, the rate of graft rejection after nDSAEK has not yet been reported. Herein, we reported for the first time that the rate of graft rejection following nDSAEK was almost identical to that after DSAEK by a HR similar to that of the multivariate model without eye drops (DSAEK: 0.57, nDSAEK: 0.56) (Table 7). This result was also confirmed by the group comparison shown in Fig. 1 (DSAEK vs. nDSAEK, *P* = 0.829), indicating that the presence of host Descemet’s membrane showed no difference between nDSAEK and DSAEK in terms of rejection.

In this study, signs of immunological rejection by slit-lamp biomicroscopic observation at diagnosis in PK, DSAEK, nDSAEK, and DMEK included conjunctival hyperemia (27.3%, 30.0%, 18.2%, and 0%, respectively), diffuse corneal edema (45.5%, 45.0%, 45.5%, and 100%, respectively), and keratic precipitates (84.8%, 95.0%, 90.9%, and 100%, respectively) (Table 5). A rejection line was observed in four cases of PK; however, no endothelial rejection lines were observed in other types of keratoplasty. Contrarily, the symptomatic rejection rates in PK, DSAEK, nDSAEK, and DMEK were 51.5%, 40.0%, 63.6%, and 100%, respectively. Collectively, careful observation using slit lamp biomicrography to detect corneal edema and keratic precipitates is crucial in detecting the early phase of graft rejection. Routine examination of corneal thickness using pachymetry and anterior segment optical coherence tomography may also help to detect subclinical graft rejection. Considering half of the rejection cases were asymptomatic, ophthalmologists should look out for signs of rejection during routine postoperative examinations.

In this study, we investigated the risk factors for graft rejection (Table 7). In the univariate model, surgical procedure, age, indications (failed keratoplasty and infection), and eye drops at the observational endpoint (rejection or last visit) had significant effects on rejection episodes, of which the most influential factor was the surgery type. The multivariate model with baseline characteristics without considering steroid eye drops usage, surgical procedure, younger age, and indications (failed keratoplasty and infection) remained after backward variable selection. In the multivariate model with eye drops (i.e., considering steroid eye drops usage), significant effects of eye drop at rejection or last visit (*P* = 0.019) were observed; steroid eye drop users had a significant risk of graft rejection compared to non-users of eye drops (i.e., fluorometholone HR: 1.37, 95% CI [0.63, 3.00] and betamethasone HR: 2.68, 95% CI [1.19, 6.04]). According to the postoperative treatment regimen in the “Methods” section, topical 0.1% betamethasone was changed to a soft steroid, such as 0.1% fluorometholone, thrice a day after 6 months. By reviewing the chart of the cases with rejection episodes, we observed use of topical 0.1% betamethasone in the following 25 cases (38.5%): PK (N = 18, 54.5%), DSAEK (N = 5, 25.0%), nDSAEK (N = 2, 18.2%), and DMEK (N = 0) (Table 5). Of these, nine cases (36%) had a history of failed keratoplasty. Furthermore, another four cases (16%) had a history of eye surgery other than cataract surgery (e.g., vitrectomy). We hypothesized that these complicated cases had a higher risk of rejection on account of having been treated with stronger steroid eye drops, such as 0.1% betamethasone. Other factors, including sex, hypertension, diabetes mellitus, herpetic keratitis, glaucoma with or without surgery, prior keratoplasty in the opposite eye, indications for keratoplasty except for failed keratoplasty and infection, combined surgery, and graft size were not identified as risk factors for graft rejection.

One limitation of this study was the difference in the background diseases of keratoplasty between Japan and other countries. In Western countries, Fuchs corneal endothelial dystrophy accounts for most of the cases of endothelial dysfunction; however, in our study, failed keratoplasty (23.6%), argon laser iridotomy (17.1%), pseudophakic bullous keratopathy (11.2%), and glaucoma surgery (10.7%) were the leading causes, and Fuchs corneal endothelial dystrophy (6.6%) was relatively rare. Nonetheless, this study is the largest keratoplasty case series in Japan and may contribute to the understanding of graft rejection in keratoplasty, which remains a severe challenge for ophthalmologists.

In conclusion, our study revealed that the type of keratoplasty was the most significant factor for graft rejection in our cohort. PK showed the highest rejection rate, followed by nDSAEK and DSAEK. Additionally, DMEK showed a relatively low incidence rate of graft rejection, confirming the superiority of this procedure over other types of keratoplasty. The presence of the host Descemet’s membrane showed no difference between the nDSAEK and DSAEK groups in terms of rejection episodes. Considering that patients who are asymptomatic exist in about half of the rejection cases, ophthalmologists should be aware of the diagnosis of rejection episodes during routine postoperative examinations.

## Methods

### Study design and patient approval

This retrospective chart review was approved by the Ethical Committee of Kanazawa University Graduate School of Medical Science (approval number: 3483–1), and the work was compliant with the Health Insurance Portability and Accountability Act of 1996 and consistent with Good Clinical Practices. It adhered to the tenets of the Declaration of Helsinki, and written informed consent was obtained from all the patients.

Anonymized data from consecutive cases of all types of corneal transplantation, including PK, anterior lamellar keratoplasty (ALK), DALK, DSAEK, nDSAEK, and DMEK, in two participating centers in Japan (Kanazawa University Hospital and Yokohama Minami Kyosai Hospital) between January 2006 and December 2020 were collected. However, cases of ALK (N = 6) and DALK (N = 33) were excluded from this study; statistical analysis was impossible due to the small sample size. Cases with patients younger than 40 years old were excluded from this study because these cases were relatively rare in Japan and sometimes quite complicated with multiple surgical histories, which may lead to misinterpretation of the whole data. Cases without chart information regarding the type of eye drop at the time of rejection or the final follow-up visit were also excluded. Additionally, cases with less than 3 months (90 days) of follow-up data were excluded, leaving 730 cases for the analysis of 566 patients. The medical records of these patients were reviewed. Notably, data, including recipient age, sex, diagnosis, types of keratoplasties, surgical histories, duration of follow-up, simultaneous surgeries at the time of keratoplasty, systemic comorbidities (hypertension, diabetes, and atopic dermatitis), ophthalmic histories (previous herpetic keratitis, pre-existing glaucoma, and prior keratoplasty in opposite eyes), clinical symptoms, final outcome regarding failure or success of the graft, and types of steroidal medications at the time of rejection or final follow-up visit, were analyzed. Moreover, slit-lamp presentations, including conjunctival hyperemia, corneal edema, keratic precipitates, vascularization of the cornea, peripheral anterior synechia, and rejection line, were recorded for patients with graft rejection. Graft failure was defined as an irreversible loss of central corneal clarity with stromal thickening from any cause with or without an endothelial rejection line, inflammation including keratic precipitates, cells in the stroma, and an increase in aqueous cells from a previous visit.

For the algorithm used to choose between nDSAEK/DSAEK and DMEK, nDSAEK/DSAEK was the preferred procedure in earlier cases performed between 2006 to 2010, when DMEK was not yet available. Also, nDSAEK/DSAEK was chosen in cases with complex eyes, such as too thick cornea, poor iris visibility, too shallow anterior chamber, and iris defect. DMEK was selected for Fuchs corneal endothelial dystrophy and other types of endothelial dysfunction with good iris visibility and sound posterior capsule.

### Surgical techniques

Three trained surgeons performed the keratoplasty surgeries at Kanazawa University Hospital (AK and HY) and Yokohama Minami Kyosai Hospital (TH). PK was performed conventionally with a 10–0 nylon suture using either a running suture or an interrupted suture technique. In most cases, a corneal trephine with a diameter of 7.5 mm with a 7.75-mm donor punch was used for PK. DSAEK was performed as previously described using a double glide (Busin glide [Moria, Antony, France] and intraocular lens sheet glide) donor insertion technique^37^ or NS Endo-Inserter (HOYA Co., Ltd, Tokyo Japan)^38^. Domestic eye bank donors dissected with a microkeratome (ALTK Cbm, Moria Japan KK, Tokyo, Japan) or precut DSAEK donors internationally shipped from a US eye bank (CorneaGen Inc., Seattle, WA, USA) with a diameter of 8.0 mm were used in most cases. For nDSAEK, neither Descemet’s membrane scoring in a circular pattern nor Descemet’s membrane stripping was performed. DMEK was performed as previously described using a pre-stripped pre-punched pre-loaded donor from a US eye bank (CorneaGen Inc., Seattle, WA, USA) with a diameter of 8.0 mm^39–43^.

### Postoperative treatment regimen

Postoperatively, topical 0.5% levofloxacin and topical 0.1% betamethasone were applied five times daily for 1 week, then tapered to four times daily for 6 months, provided they did not indicate steroid-induced glaucoma. After 6 months, topical 0.1% betamethasone was changed to soft steroids, including 0.1% fluorometholone, thrice daily. Moreover, following 1 year, the eyes were usually maintained on once-daily steroid dosing indefinitely as long as no signs or symptoms of graft rejection occurred. In eyes with steroid-induced intraocular pressure (IOP) control problems, topical 0.1% betamethasone was usually changed to a soft steroid, such as 0.1% fluorometholone, and dosing was adjusted as needed to adequately lower IOP. Glaucoma agents were added if necessary. For cases of endothelial keratoplasties, including DSAEK, nDSAEK, and DMEK, 0.1% bromfenac eyedrop two times daily was used for 1 month to prevent postoperative cystoid macular edema.

At the time of a rejection episode, topical 0.1% betamethasone was prescribed eight times daily, approximately hourly while awake and oral prednisolone was added in cases where graft rejection seemed severe. The dosage of topical 0.1% betamethasone was maintained for 1 week and gradually tapered to 3–4 times a day for several months. In cases with severe rejection, oral prednisolone or intravenous administration of betamethasone was used.

### Statistical analysis

Demographics and other baseline characteristics were summarized with appropriate descriptive statistics, such as mean (SD), by the surgical procedure group, PK, DSAEK, nDSAEK, and DMEK groups. Eye drops at rejection or the last visit and graft outcomes were also summarized. In this analysis, the date rejection was first diagnosed was recorded as the date of the “event” or eyes that did not experience a rejection episode, and the date of the most recent follow-up exam with no evidence of rejection was used to determine the length of follow-up. The findings and graft outcomes were summarized for cases with a rejection episode. To evaluate heterogeneity in demographics and other characteristics among surgical procedures, the Kruskal–Wallis test for continuous variables and Chi-square test for categorical variables were used. Additionally, the incidence rate of rejection was calculated as the time interval after keratoplasty using the person-year method. The time to rejection from keratoplasty was graphically described by the surgical procedure group using the Kaplan–Meier curve. HRs between the groups were estimated using Cox regression, and the curves were compared using the log-rank test. Univariate and multivariate Cox proportional hazards regressions were applied to detect risk factors for rejection episodes. Moreover, the multivariate model with baseline characteristics included surgical procedure, age, sex, hypertension, diabetes mellitus, herpetic keratitis, glaucoma with or without surgery, prior keratoplasty in the opposite eye, indications for keratoplasty, combined surgery, and graft size as exploratory variables, which were selected by backward elimination. Eye drops at rejection or the last visit were included in the multivariate model with baseline characteristics.

Statistical significance was set at *P* < 0.05. Statistical analyses were performed using R, version 4.2.1^44^.

## Supplementary Information

Supplementary Information.

## Data Availability

The authors declare that all data generated or analyzed during this study are included in this published article and its supplementary information files.
