# Supplementary material for: Graft rejection episodes after keratoplasty in Japanese eyes
Source: Sci Rep. 2023 Feb 14;13:2635. doi: 10.1038/s41598-023-29659-w (PMC9929100; doi:10.1038/s41598-023-29659-w)
Supplement: Supplementary file 1 — Supplementary Information. [file 41598_2023_29659_MOESM1_ESM.docx]

### Table S1. Rejection episodes by demographics and comorbidities

| Characteristics | Total N = 730 | | [A] PK N = 198 | | [B] DSAEK N = 277 | | [C] nDSAEK N = 138 | | [D] DMEK N = 117 | |
| --- | --- | --- | --- | --- | --- | --- | --- | --- | --- | --- |
|  | n | Rejection | n | Rejection | n | Rejection | n | Rejection | n | Rejection |
| Age |  |  |  |  |  |  |  |  |  |  |
| [1] 40-69 years | 253 | 33 (13.0%) | 98 | 18 (18.4%) | 89 | 6 (6.7%) | 38 | 8 (21.1%) | 28 | 1 (3.6%) |
| [2] 70-97 years | 477 | 32 (6.7%) | 100 | 15 (15.0%) | 188 | 14 (7.4%) | 100 | 3 (3.0%) | 89 | 0 (0.0%) |
| Sex | | | | | | | | | | |
| [1] Male | 359 | 36 (10.0%) | 119 | 20 (16.8%) | 145 | 12 (8.3%) | 56 | 4 (7.1%) | 39 | 0 (0.0%) |
| [2] Female | 371 | 29 (7.8%) | 79 | 13 (16.5%) | 132 | 8 (6.1%) | 82 | 7 (8.5%) | 78 | 1 (1.3%) |
| Hypertension | | | | | | | | | | |
| [1] No | 581 | 54 (9.3%) | 166 | 27 (16.3%) | 208 | 17 (8.2%) | 102 | 9 (8.8%) | 105 | 1 (1.0%) |
| [2] Yes | 149 | 11 (7.4%) | 32 | 6 (18.8%) | 69 | 3 (4.3%) | 36 | 2 (5.6%) | 12 | 0 (0.0%) |
| Diabetes mellitus | | | | | | | | | | |
| [1] No | 631 | 56 (8.9%) | 177 | 29 (16.4%) | 225 | 17 (7.6%) | 120 | 9 (7.5%) | 109 | 1 (0.9%) |
| [2] Yes | 99 | 9 (9.1%) | 21 | 4 (19.0%) | 52 | 3 (5.8%) | 18 | 2 (11.1%) | 8 | 0 (0.0%) |
| Atopic dermatitis | | | | | | | | | | |
| [1] No | 719 | 65 (9.0%) | 193 | 33 (17.1%) | 271 | 20 (7.4%) | 138 | 11 (8.0%) | 117 | 1 (0.9%) |
| [2] Yes | 11 | 0 (0.0%) | 5 | 0 (0.0%) | 6 | 0 (0.0%) | 0 | - | 0 | - |

Continued onto next page

| Characteristics | Total N = 730 | | [A] PK N = 198 | | [B] DSAEK N = 277 | | [C] nDSAEK N = 138 | | [D] DMEK N = 117 | |
| --- | --- | --- | --- | --- | --- | --- | --- | --- | --- | --- |
|  | n | Rejection | n | Rejection | n | Rejection | n | Rejection | n | Rejection |
| Herpetic keratitis | | | | | | | | | | |
| [1] No | 688 | 59 (8.6%) | 171 | 28 (16.4%) | 268 | 20 (7.5%) | 133 | 10 (7.5%) | 116 | 1 (0.9%) |
| [2] Yes | 42 | 6 (14.3%) | 27 | 5 (18.5%) | 9 | 0 (0.0%) | 5 | 1 (20.0%) | 1 | 0 (0.0%) |
| Glaucoma and surgery | | | | | | | | | | |
| [1] No glaucoma | 514 | 45 (8.8%) | 150 | 28 (18.7%) | 158 | 9 (5.7%) | 98 | 7 (7.1%) | 108 | 1 (0.9%) |
| [2] Glaucoma without surgery | 64 | 3 (4.7%) | 14 | 2 (14.3%) | 27 | 1 (3.7%) | 15 | 0 (0.0%) | 8 | 0 (0.0%) |
| [3] Glaucoma with surgery | 152 | 17 (11.2%) | 34 | 3 (8.8%) | 92 | 10 (10.9%) | 25 | 4 (16.0%) | 1 | 0 (0.0%) |
| Prior keratoplasty in opposite eye | | | | | | | | | | |
| [1] No | 572 | 46 (8.0%) | 158 | 23 (14.6%) | 222 | 14 (6.3%) | 113 | 8 (7.1%) | 79 | 1 (1.3%) |
| [2] Yes | 157 | 19 (12.1%) | 39 | 10 (25.6%) | 55 | 6 (10.9%) | 25 | 3 (12.0%) | 38 | 0 (0.0%) |
| [3] Unknown | 1 | 0 (0.0%) | 1 | 0 (0.0%) | 0 | - | 0 | - | 0 | - |
| Filtering bleb |  |  |  |  |  |  |  |  |  |  |
| [1] No | 603 | 51 (8.5%) | 173 | 30 (17.3%) | 197 | 12 (6.1%) | 116 | 8 (6.9%) | 117 | 1 (0.9%) |
| [2] Yes | 127 | 14 (11.0%) | 25 | 3 (12.0%) | 80 | 8 (10.0%) | 22 | 3 (13.6%) | 0 | - |

DMEK, Descemet’s membrane endothelial keratoplasty; (n)DSAEK, (Non-)Descemet’s stripping automated endothelial keratoplasty; PK, penetrating keratoplasty.

### Table S2. Rejection episodes by indication of keratoplasty

| Characteristics | Total N = 730 | | [A] PK N = 198 | | [B] DSAEK N = 277 | | [C] nDSAEK N = 138 | | [D] DMEK N = 117 | |
| --- | --- | --- | --- | --- | --- | --- | --- | --- | --- | --- |
|  | n | Rejection | n | Rejection | n | Rejection | n | Rejection | n | Rejection |
| Indication |  |  |  |  |  |  |  |  |  |  |
| [01] Failed keratoplasty | 172 | 23 (13.4%) | 44 | 10 (22.7%) | 95 | 9 (9.5%) | 26 | 4 (15.4%) | 7 | 0 (0.0%) |
| [02] ALI | 125 | 8 (6.4%) | 4 | 2 (50.0%) | 33 | 1 (3.0%) | 47 | 4 (8.5%) | 41 | 1 (2.4%) |
| [03] PBK | 82 | 3 (3.7%) | 6 | 1 (16.7%) | 34 | 1 (2.9%) | 26 | 1 (3.8%) | 16 | 0 (0.0%) |
| [04] Glaucoma surgery | 78 | 6 (7.7%) | 14 | 0 (0.0%) | 48 | 4 (8.3%) | 16 | 2 (12.5%) | 0 | - |
| [05] Corneal opacity | 48 | 8 (16.7%) | 40 | 7 (17.5%) | 7 | 1 (14.3%) | 0 | - | 1 | 0 (0.0%) |
| [06] FED | 48 | 2 (4.2%) | 0 | - | 17 | 2 (11.8%) | 3 | 0 (0.0%) | 28 | 0 (0.0%) |
| [07] XFS | 27 | 1 (3.7%) | 1 | 0 (0.0%) | 11 | 1 (9.1%) | 6 | 0 (0.0%) | 9 | 0 (0.0%) |
| [08] Keratoconus | 21 | 4 (19.0%) | 21 | 4 (19.0%) | 0 | - | 0 | - | 0 | - |
| [09] Perforation | 17 | 1 (5.9%) | 17 | 1 (5.9%) | 0 | - | 0 | - | 0 | - |
| [10] Infection | 13 | 4 (30.8%) | 13 | 4 (30.8%) | 0 | - | 0 | - | 0 | - |
| [11] CMV corneal endotheliitis | 12 | 0 (0.0%) | 0 | - | 7 | 0 (0.0%) | 1 | 0 (0.0%) | 4 | 0 (0.0%) |
| [12] Corneal dystrophy/degeneration | 11 | 1 (9.1%) | 7 | 1 (14.3%) | 1 | 0 (0.0%) | 0 | - | 3 | 0 (0.0%) |
| [13] ICE | 3 | 0 (0.0%) | 1 | 0 (0.0%) | 2 | 0 (0.0%) | 0 | - | 0 | - |
| [14] Others | 73 | 4 (5.5%) | 30 | 3 (10.0%) | 22 | 1 (4.5%) | 13 | 0 (0.0%) | 8 | 0 (0.0%) |

Abbreviations: ALI, argon laser iridotomy; CMV, cytomegalovirus; DMEK, Descemet’s membrane endothelial keratoplasty; (n)DSAEK, (non-)Descemet’s stripping automated endothelial keratoplasty; FED, Fuchs’ endothelial dystrophy; ICE, iridocorneal endothelial syndrome; PBK, pseudophakic bullous keratopathy; PK, penetrating keratoplasty; XFS, exfoliation syndrome.

###

### Table S3. Rejection episodes by simple/combined keratoplasty and graft size

| Characteristics | Total N = 730 | | [A] PK N = 198 | | [B] DSAEK N = 277 | | [C] nDSAEK N = 138 | | [D] DMEK N = 117 | |
| --- | --- | --- | --- | --- | --- | --- | --- | --- | --- | --- |
|  | n | Rejection | n | Rejection | n | Rejection | n | Rejection | n | Rejection |
| Simple vs. Combined keratoplasty |  |  |  |  |  |  |  |  |  |  |
| [1] Simple keratoplasty | 603 | 56 (9.3%) | 160 | 27 (16.9%) | 230 | 18 (7.8%) | 113 | 10 (8.8%) | 100 | 1 (1.0%) |
| [2] Combined keratoplasty | 127 | 9 (7.1%) | 38 | 6 (15.8%) | 47 | 2 (4.3%) | 25 | 1 (4.0%) | 17 | 0 (0.0%) |
| Graft size |  |  |  |  |  |  |  |  |  |  |
| [1] 4.0-<7.6 mm | 195 | 23 (11.8%) | 66 | 12 (18.2%) | 79 | 7 (8.9%) | 36 | 3 (8.3%) | 14 | 1 (7.1%) |
| [2] 7.6-<7.8 mm | 128 | 18 (14.1%) | 105 | 17 (16.2%) | 4 | 0 (0.0%) | 5 | 1 (20.0%) | 14 | 0 (0.0%) |
| [3] 7.8-<8.1 mm | 316 | 16 (5.1%) | 18 | 2 (11.1%) | 155 | 9 (5.8%) | 80 | 5 (6.2%) | 63 | 0 (0.0%) |
| [4] 8.1-<9.0 mm | 57 | 5 (8.8%) | 5 | 0 (0.0%) | 27 | 4 (14.8%) | 6 | 1 (16.7%) | 19 | 0 (0.0%) |
| [5] Unknown | 34 | 3 (8.8%) | 4 | 2 (50.0%) | 12 | 0 (0.0%) | 11 | 1 (9.1%) | 7 | 0 (0.0%) |

DMEK, Descemet’s membrane endothelial keratoplasty; (n)DSAEK, (Non-)Descemet’s stripping automated endothelial keratoplasty; PK, penetrating keratoplasty.
